# Tobacco Product Use Among Adults — United States, 2015

**DOI:** 10.15585/mmwr.mm6644a2

**Published:** 2017-11-10

**Authors:** Elyse Phillips, Teresa W. Wang, Corinne G. Husten, Catherine G. Corey, Benjamin J. Apelberg, Ahmed Jamal, David M. Homa, Brian A. King

**Affiliations:** ^1^Office on Smoking and Health, National Center for Chronic Disease Prevention and Health Promotion, CDC; ^2^Epidemic Intelligence Service, CDC; ^3^Center for Tobacco Products, Food and Drug Administration.

Tobacco use remains the leading cause of preventable disease and death in the United States (*1*). Despite declining cigarette smoking prevalence among U.S. adults, shifts in the tobacco product landscape have occurred in recent years (*2*,*3*). Previous estimates of tobacco product use among U.S. adults were obtained from the National Adult Tobacco Survey, which ended after the 2013–2014 cycle. This year, CDC and the Food and Drug Administration (FDA) assessed the most recent national estimates of tobacco product use among adults aged ≥18 years using, for the first time, data from the 2015 National Health Interview Survey (NHIS), an annual, nationally representative, in-person survey of the noninstitutionalized U.S. civilian population. The 2015 NHIS adult core questionnaire included 33,672 adults aged ≥18 years, reflecting a 55.2% response rate. Data were weighted to adjust for differences in selection probability and nonresponse, and to provide nationally representative estimates. In 2015, 20.1 % of U.S. adults currently (every day or some days) used any tobacco product, 17.6% used any combustible tobacco product, and 3.9% used ≥2 tobacco products. By product, 15.1% of adults used cigarettes; 3.5% used electronic cigarettes (e-cigarettes); 3.4% used cigars, cigarillos, or filtered little cigars; 2.3% used smokeless tobacco; and 1.2% used regular pipes, water pipes, or hookahs.* Current use of any tobacco product was higher among males; persons aged <65 years; non-Hispanic American Indian/Alaska natives (AI/AN), whites, blacks, and persons of multiple races; persons living in the Midwest; persons with a General Educational Development (GED) certificate; persons with annual household income of <$35,000; persons who were single, never married, or not living with a partner or divorced, separated, or widowed; persons who were insured through Medicaid or uninsured; persons with a disability; and persons who identified as lesbian, gay, or bisexual (LGB). Current use of any tobacco product was 47.2% among adults with serious psychological distress compared with 19.2% among those without serious psychological distress. Proven population-level interventions that focus on the diversity of tobacco product use are important to reducing tobacco-related disease and death in the United States (*1*).

Consistent with previous reports (*2*,*3*), current cigarette smokers were defined as persons who reported they had smoked ≥100 cigarettes during their lifetime, and smoked either “every day” or “some days” at the time of survey. Current users of all other assessed tobacco products were defined as persons who reported use “every day” or “some days” at the time of survey. Prevalence estimates for current use of any current tobacco product, any combustible tobacco product (cigarettes, cigars, cigarillos, filtered little cigars, pipes, water pipes, or hookahs), and use of two or more tobacco products were calculated. Estimates were assessed overall and by sex, age, race/ethnicity, U.S. Census region,^†^ education, marital status, annual household income, sexual orientation,^§^ health insurance coverage,^¶^ disability,** and presence of serious psychological distress.^††^ Significant differences between groups were assessed using chi-squared statistics; differences presented were all statistically significant (p<0.05).

Among U.S. adults in 2015, 20.1% (an estimated 48.7 million) currently used any tobacco product, 17.6% (42.6 million; 87.4% of current tobacco product users) currently used any combustible tobacco product, and 3.9% (9.5 million; 19.5%) currently used ≥2 tobacco products. By product, 15.1% (36.5 million; 74.9% of current users) of adults currently used cigarettes; 3.5% (7.9 million; 16.1%) used e-cigarettes; 3.4% (7.8 million; 16.0%) used cigars, cigarillos, or filtered little cigars; 2.3% (5.1 million; 10.5%) used smokeless tobacco; and 1.2% (2.7 million; 5.5%) used pipes, water pipes, or hookahs.

Differences in tobacco product use were observed across population groups (Table). The prevalence of any current tobacco use was significantly higher among males (25.2%) than among females (15.4%) and among adults aged 25–44 years (23.3%) than among those aged ≥65 years (11.1%). Notably, the age distribution of current tobacco users varied by product type, and for pipes, water pipes, hookahs and e-cigarettes, use was highest among younger adults (Figure). By race/ethnicity, current use was higher among non-Hispanic AI/AN (26.6%), multiple races (25.4%), whites (22.6%), and blacks (20.8%), and lowest among non-Hispanic Asians (9.0%). By region, prevalence was highest among adults living in the Midwest (24.0%) and lowest among those living in the West (17.4%). Prevalence was highest among adults with a GED certificate (37.6%) and lowest among those with a graduate degree (6.9%), and was higher among adults who were single, never married, or not living with a partner (23.1%) or divorced, separated, or widowed (23.2%) than among adults who were married or living with a partner (18.2%). Prevalence of tobacco use was highest among persons with an income of <$35,000 (27.8%) and lowest among those with an annual household income of ≥$100,000 (13.4%); it was also higher among LGB adults (27.4%) than among heterosexual adults (20.1%), and among uninsured persons (32.3%) and Medicaid enrollees (31.7%) than among those covered by private health insurance (16.6%) or by Medicare only (11.4%). Adults with a disability had higher prevalence (25.8%) of tobacco use than did those reporting no disability (19.7%), and prevalence was higher among adults with serious psychological distress (47.2%) than adults without serious psychological distress (19.2%).

**TABLE:** Percentage of persons aged ≥18 years who reported tobacco product use "every day" or "some days," by tobacco product and selected characteristics — National Health Interview Survey, United States, 2015

Characteristic	Tobacco product use, % (95% CI)							
	Any tobacco product*	Any combustible tobacco product^†^	Cigarettes^§^	Cigars/Cigarillos/Filtered little cigars^¶^	Regular pipe/Water pipe/Hookah**	E-cigarettes^††^	Smokeless tobacco^§§^	≥2 tobacco products^¶¶^
**Overall**	20.1 (19.5–20.8)	17.6 (17.0–18.2)	15.1 (14.6–15.7)	3.4 (3.1–3.7)	1.2 (1.0–1.4)	3.5 (3.2–3.8)	2.3 (2.0–2.6)	3.9 (3.6–4.2)
**Sex**								
Male	25.2 (24.2–26.3)	21.0 (20.1–22.0)	16.7 (15.9–17.6)	6.0 (5.4–6.5)	1.8 (1.5–2.2)	4.3 (3.9–4.8)	4.4 (3.9–5.0)	5.8 (5.3–6.3)
Female	15.4 (14.7–16.1)	14.4 (13.8–15.1)	13.6 (12.9–14.3)	1.1 (0.9–1.3)	0.6 (0.4–0.8)	2.6 (2.3–3.0)	0.2 (0.1–0.3)	2.2 (1.9–2.5)
**Age group (yrs)**								
18–24	21.4 (19.3–23.5)	17.6 (15.8–19.5)	13.0 (11.4–14.8)	4.2 (3.3–5.3)	3.4 (2.6–4.4)	5.2 (4.3–6.3)	3.2 (2.4–4.3)	5.4 (4.4–6.7)
25–44	23.3 (22.2–24.5)	20.3 (19.3–21.4)	17.7 (16.8–18.8)	3.9 (3.4–4.5)	1.3 (1.0–1.7)	4.3 (3.8–4.9)	2.7 (2.3–3.1)	4.8 (4.2–5.4)
45–64	21.6 (20.5–22.7)	19.2 (18.2–20.3)	17.0 (16.0–18.0)	3.7 (3.2–4.2)	0.5 (0.4–0.8)	3.3 (2.8–3.7)	2.1 (1.7–2.5)	3.9 (3.5–4.4)
≥65	11.1 (10.2–12.0)	9.8 (9.0–10.7)	8.4 (7.7–9.2)	1.7 (1.4–2.1)	0.6 (0.4–0.9)	1.1 (0.8–1.5)	1.2 (0.9–1.7)	1.5 (1.2–1.9)
**Race/Ethnicity**								
White, non-Hispanic	22.6 (21.7–23.5)	19.3 (18.5–20.1)	16.6 (15.8–17.40)	3.7 (3.3–4.1)	1.2 (1.0–1.4)	4.1 (3.7–4.6)	3.2 (2.8–3.6)	4.6 (4.2–5.1)
Black, non-Hispanic	20.8 (19.1–22.6)	19.9 (18.2–21.6)	16.7 (15.2–18.3)	4.8 (3.9–5.7)	1.4 (1.0–2.1)	1.9 (1.4–2.5)	0.7 (0.5–1.0)	3.7 (3.1–4.6)
Asian, non-Hispanic	9.0 (7.5–10.8)	8.0 (6.7–9.7)	7.0 (5.7–8.6)	0.9 (0.5–1.6)	—***	2.3 (1.4–3.6)	—***	1.5 (0.9–2.4)
American Indian/Alaska Native, non-Hispanic	26.6 (20.1–34.4)	24.8 (18.3–32.6)	21.9 (17.0–27.6)	—***	—***	—***	—***	—***
Hispanic	12.9 (11.8–14.1)	11.8 (10.8–12.9)	10.1 (9.1–11.1)	1.9 (1.5–2.5)	0.8 (0.5–1.1)	2.0 (1.5–2.5)	0.4 (0.2–0.6)	1.6 (1.3–2.0)
Non-Hispanic multirace	25.4 (21.3–29.9)	23.6 (19.6–28.1)	20.2 (16.3–24.8)	6.8 (4.4–10.3)	—***	7.1 (4.2–11.8)	—***	9.3 (6.6–13.0)
**U.S. Census region^†††^**								
Northeast	18.2 (16.7–19.9)	16.6 (15.1–18.2)	13.5 (12.3–14.9)	3.8 (2.9–4.8)	1.3 (0.9–1.9)	2.6 (1.9–3.4)	1.1 (0.7–1.6)	3.1 (2.4–4.1)
Midwest	24.0 (22.6–25.5)	21.1 (19.8–22.4)	18.7 (17.4–20.1)	3.7 (3.1–4.4)	1.1 (0.8–1.6)	3.8 (3.2–4.5)	3.1 (2.4–4.0)	4.7 (4.0–5.5)
South	20.4 (19.4–21.6)	17.5 (16.6–18.4)	15.3 (14.5–16.3)	3.3 (3.0–3.8)	0.9 (0.7–1.2)	3.5 (3.1–4.0)	2.7 (2.3–3.2)	3.9 (3.5–4.4)
West	17.4 (16.3–18.5)	15.1 (14.1–16.2)	12.4 (11.4–13.5)	3.1 (2.5–3.8)	1.5 (1.2–2.0)	3.7 (3.2–4.3)	1.6 (1.2–2.1)	3.7 (3.1–4.4)
**Education (results are adults aged ≥25 yrs)**								
0–12 yrs (no diploma)	27.6 (25.7–29.6)	25.0 (23.2–26.9)	24.2 (22.5–26.1)	3.0 (2.2–4.0)	1.2 (0.7–2.0)	3.3 (2.5–4.3)	2.9 (2.2–3.9)	5.0 (4.0–6.2)
GED	37.6 (33.3–42.3)	35.9 (31.7–40.3)	34.1 (30.0–38.4)	4.7 (3.2–7.0)	—***	6.3 (4.6–8.5)	2.6 (1.6–4.2)	8.5 (6.6–10.9)
High school diploma	24.4 (22.8–26.0)	21.4 (20.0–22.9)	19.8 (18.5–21.2)	3.4 (2.8–4.2)	0.6 (0.4–0.9)	3.6 (3.0–4.4)	2.8 (2.2–3.5)	4.5 (3.9–5.3)
Some college, no degree	23.8 (22.2–25.3)	20.5 (19.2–21.9)	18.5 (17.2–19.8)	3.3 (2.7–4.1)	0.7 (0.5–1.1)	4.6 (3.8–5.6)	2.2 (1.8–2.9)	4.4 (3.7–5.2)
Associate degree (academic or technical/vocational)	22.2 (20.4–24.1)	19.4 (17.8–21.2)	16.6 (15.0–18.3)	3.9 (3.1–4.9)	1.0 (0.6–1.5)	4.2 (3.3–5.2)	2.5 (1.7–3.8)	4.5 (3.5–5.8)
Undergraduate degree (BA, BS, AB, BBA)	12.6 (11.5–13.8)	10.6 (9.6–11.7)	7.4 (6.5–8.3)	3.4 (2.8–4.2)	1.2 (0.8–1.7)	2.4 (1.9–3.0)	1.5 (1.1–2.0)	2.4 (1.9–2.0)
Graduate degree (Master's, Professional, or Doctoral)	6.9 (5.9–8.0)	6.3 (5.4–7.4)	3.6 (3.0–4.5)	2.5 (1.9–3.4)	0.7 (0.4–1.1)	0.6 (0.4–1.0)	0.7 (0.4–1.2)	0.9 (0.6–1.4)
**Marital status**								
Married/living with partner	18.2 (17.3–19.1)	15.5 (14.8–16.3)	13.1 (12.4–13.9)	3.3 (2.9–3.7)	0.7 (0.5–0.9)	3.1 (2.8–3.5)	2.3 (2.0–2.7)	3.3 (2.9–3.7)
Divorced/Separated/Widowed	23.2 (22.0–24.6)	21.3 (20.1–22.5)	20.0 (18.8–21.2)	2.8 (2.3–3.4)	0.8 (0.6–1.1)	3.1 (2.6–3.6)	2.1 (1.6–2.7)	4.3 (3.7–5.0)
Single/Never married/Not living with a partner	23.1 (21.8–24.6)	20.3 (19.1–21.6)	16.6 (15.4–17.9)	4.4 (3.8–5.2)	2.9 (2.3–3.6)	4.7 (4.0–5.5)	2.3 (1.8–2.9)	5.4 (4.7–6.1)
**Annual household income ($)**								
<35,000	27.8 (26.6–29.0)	25.4 (24.2–26.6)	23.3 (22.2–24.5)	3.8 (3.4–4.3)	1.6 (1.3–2.1)	4.6 (4.1–5.2)	2.1 (1.7–2.6)	5.8 (5.2–6.4)
35,000–74,999	21.2 (20.0–22.5)	18.6 (17.5–19.8)	16.6 (15.6–17.8)	2.9 (2.4–3.4)	1.2 (0.9–1.6)	3.5 (3.0–4.1)	2.3 (1.9–2.9)	3.9 (3.4–4.6)
75,000–99,999	18.1 (16.3–20.2)	14.7 (13.0–16.5)	11.9 (10.5–13.4)	3.7 (2.7–4.9)	—***	4.2 (3.2–5.3)	2.7 (1.9–3.8)	3.8 (2.9–5.0)
≥100,000	13.4 (12.3–14.7)	10.9 (9.8–12.1)	7.1 (6.2–8.2)	3.8 (3.1–4.5)	1.2 (0.8–1.7)	2.3 (1.8–2.8)	2.3 (1.8–2.9)	2.3 (1.8–2.9)
**Sexual orientation**								
Heterosexual/Straight	20.1 (19.4–20.8)	17.5 (16.9–18.1)	14.9 (14.4–15.5)	3.4 (3.1–3.7)	1.1 (0.9–1.3)	3.4 (3.1–3.7)	2.3 (2.0–2.6)	3.9 (3.6–4.3)
LGB	27.4 (23.5–31.7)	24.3 (20.5–28.4)	20.6 (17.1–24.6)	3.8 (2.4–5.8)	4.0 (2.5–6.2)	8.9 (6.5–11.9)	—***	7.6 (5.6–10.2)
**Health insurance coverage^§§§^**								
Private insurance	16.6 (15.8–17.4)	13.8 (13.1–14.6)	11.1 (10.5–11.8)	3.2 (2.9–3.6)	1.1 (0.9–1.3)	2.9 (2.6–3.3)	2.4 (2.0–2.8)	3.0 (2.7–3.4)
Medicaid	31.7 (29.8–33.7)	29.4 (27.6–31.3)	27.8 (26.0–29.7)	4.0 (3.2–4.9)	1.5 (1.0–2.2)	5.7 (4.7–6.8)	1.6 (1.1–2.4)	6.7 (5.7–7.9)
Medicare only (aged ≥65 yrs)	11.4 (9.9–13.1)	10.2 (8.8–11.8)	8.9 (7.6–10.5)	1.5 (1.0–2.1)	0.7 (0.4–1.3)	1.2 (0.8–1.9)	1.0 (0.6–1.7)	1.6 (1.1–2.4)
Other public insurance	25.4 (22.6–28.4)	21.9 (19.4–24.7)	19.0 (16.8–21.4)	4.7 (3.4–6.4)	—***	5.0 (3.9–6.4)	2.8 (2.0–4.1)	6.0 (4.8–7.5)
Uninsured	32.3 (30.1–34.5)	30.1 (28.0–32.2)	27.4 (25.5–29.4)	4.7 (3.8–5.9)	1.4 (1.0–2.1)	5.1 (4.2–6.2)	2.4 (1.8–3.3)	6.5 (5.5–7.7)
**Disability/Limitation^¶¶¶^**								
Yes	25.8 (23.9–27.8)	23.4 (21.6–25.4)	22.0 (20.2–24.0)	3.7 (2.9–4.6)	1.1 (0.8–1.7)	4.9 (4.0–6.1)	1.8 (1.3–2.5)	6.2 (5.2–7.4)
No	19.7 (18.8–20.6)	17.0 (16.2–17.9)	14.4 (13.7–15.2)	3.4 (3.0–3.8)	1.1 (0.9–1.3)	3.3 (2.9–3.7)	2.3 (1.9–2.7)	3.5 (3.1–3.9)
Serious psychological distress (Kessler scale)^****^								
Yes	47.2 (43.4–51.2)	43.5 (39.7–47.4)	40.6 (37.0–44.3)	6.3 (4.3–9.1)	4.3 (2.5–7.2)	9.7 (7.4–12.7)	3.5 (2.1–5.6)	12.8 (10.1–16.0)
No	19.2 (18.5–19.9)	16.6 (16.0–17.2)	14.0 (13.5–14.6)	3.3 (3.0–3.6)	1.0 (0.9–1.2)	3.2 (2.9–3.5)	2.2 (1.9–2.5)	3.7 (3.5–4.1)

**Abbreviations:** CI = confidence interval; E-cigarettes = electronic cigarettes; GED = General Education Development certificate; HS = high school; LGB = lesbian, gay, or bisexual.

* Any tobacco use was defined as use either "every day" or "some days" of at least one tobacco product among individuals (for cigarettes, users were defined as persons who reported use either "every day" or "some days" and had smoked ≥100 cigarettes during their lifetime).

^†^ Any combustible tobacco use was defined as use either "every day" or "some days" of at least one combustible tobacco product: cigarettes; cigars, cigarillos, filtered little cigars; pipes, water pipes, or hookah (for cigarettes, users were defined as persons who reported use either "every day" or "some days" and had smoked ≥100 cigarette during their lifetime).

^§^ Current cigarette smokers were defined as persons who reported smoking ≥100 cigarettes during their lifetime and now smoked cigarettes “every day” or “some days.”

^¶^ Reported smoking cigars, cigarillos, or little filtered cigars at least once during their lifetime and now smoked at least one of these products “every day” or “some days.”

** Reported smoking tobacco in a regular pipe, water pipe, or hookah at least once during their lifetime and now smoked at least one of these products “every day” or “some days.”

^††^ Reported using electronic cigarettes at least once during their lifetime and now used e-cigarettes “every day” or “some days.”

^§§^ Reported using chewing tobacco, snuff, dip, snus, or dissolvable tobacco at least once during their lifetime and now used at least one of these products “every day” or “some days.”

^¶¶^ Use was defined as use either "every day" or "some days" for at least two or more of the following tobacco products: cigarettes (≥100 cigarettes during lifetime); cigars, cigarillos, filtered little cigars; pipes, water pipes, or hookah; electronic cigarettes; or smokeless tobacco products.

*** Prevalence estimates with a relative standard error ≥30% are not presented.

^†††^ *Northeast:* Connecticut, Maine, Massachusetts, New Hampshire, New Jersey, New York, Pennsylvania, Rhode Island, and Vermont; *Midwest:* Illinois, Indiana, Iowa, Kansas, Michigan, Minnesota, Missouri, Nebraska, North Dakota, Ohio, South Dakota, and Wisconsin; *South:* Alabama, Arkansas, Delaware, District of Columbia, Florida, Georgia, Kentucky, Louisiana, Maryland, Mississippi, North Carolina, Oklahoma, South Carolina, Tennessee, Texas, Virginia, and West Virginia; *West:* Alaska, Arizona, California, Colorado, Hawaii, Idaho, Montana, Nevada, New Mexico, Oregon, Utah, Washington, and Wyoming.

^§§§^ Private coverage: includes adults who had any comprehensive private insurance plan (including health maintenance organizations and preferred provider organizations). *Medicaid*: For adults aged <65 years, includes adults who do not have private coverage, but who have Medicaid or other state-sponsored health plans including Children’s Health Insurance Program (CHIP); for adults aged ≥65 years, includes adults aged ≥65 years who do not have any private coverage but have Medicare and Medicaid or other state-sponsored health plans including CHIP. *Medicare only*: includes adults aged ≥65 years who only have Medicare coverage. *Other coverage*: includes adults who do not have private insurance, Medicaid, or other public coverage, but who have any type of military coverage, coverage from other government programs, or Medicare. *Uninsured*: includes adults who have not indicated that they are covered at the time of the interview under private health insurance, Medicare, Medicaid, CHIP, a state-sponsored health plan, other government programs, or military coverage. Insurance coverage is “as of time of survey.”

^¶¶¶^ Disability was defined based on self-reported presence of selected limitations including vision, hearing, cognition, and movement. Limitations in performing activities of daily living were defined based on response to the question, “Does [person] have difficulty dressing or bathing?” Limitations in performing instrumental activities of daily living were defined based on response to the question, “Because of a physical, mental, or emotional condition, does [person] have difficulty doing errands alone such as visiting a doctor’s office or shopping?” Any disability was defined as a “yes” response pertaining to at least one of the limitations listed (i.e., vision, hearing, cognition, movement, activities of daily living, or instrumental activities of daily living). A random sample of half of the respondents from the 2015 Person File were asked about limitations.

**** The Kessler psychological distress scale is a series of six questions that ask about feelings of sadness, nervousness, restlessness, worthlessness, and feeling like everything is an effort in the past 30 days. Participants were asked to respond on a Likert Scale ranging from “None of the time” (score = 0) to “All of the time” (score = 4). Responses were summed over the six questions; persons with a score of ≥13 were coded as having serious psychological distress, and respondents with a score <13 were coded as not having serious psychological distress.

**FIGURE:**
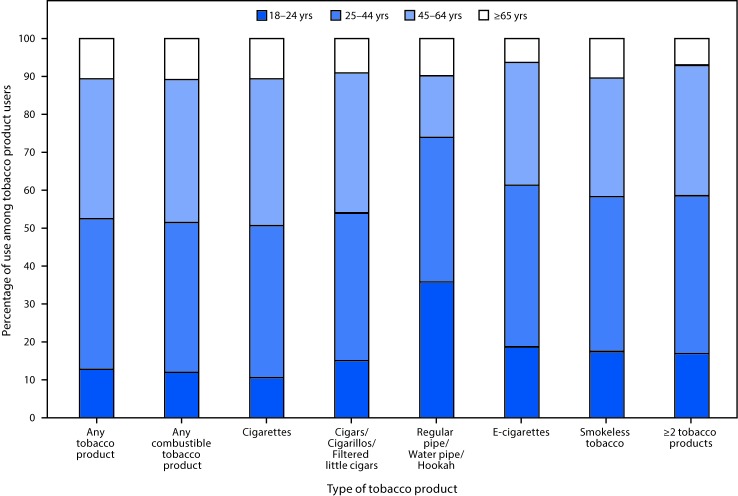
Percentage of use of tobacco product types* among adults aged ≥18 years who reported using tobacco products "every day" or "some days," by age group — National Health Interview Survey, United States, 2015 * For cigarettes, users were defined as persons who reported use either "every day" or "some days" and had smoked ≥100 cigarettes during their lifetime.

## Discussion

In 2015, approximately one in five U.S. adults (48.7 million) currently used any tobacco product, with most using combustible tobacco products. Any tobacco product use was significantly higher among males; adults aged <65 years; non-Hispanic AI/AN, whites, blacks, and persons of multiple races; persons living in the Midwest; persons with a GED; persons with annual household income <$35,000; persons who were single/never married/not living with a partner or divorced/separated/widowed; persons who were uninsured or insured through Medicaid; persons with a disability; and persons who identified as LGB. Adults with serious psychological distress had the highest prevalence of any tobacco product use of any subpopulation.

The burden of death and disease from tobacco use is overwhelmingly caused by cigarettes and other combusted tobacco products (*1*). Cigarette smoking has been declining among U.S. adults for several decades (*1*); in more recent years, prevalence declined from 20.9% in 2005 to 15.1% in 2015 (*3*). The findings from this report show that in 2015, cigarettes remained the most commonly used tobacco product among adults, and combustible tobacco products were currently used by 17.6% of adults, or 87.4% of current any tobacco users. Despite the popularity of emerging products such as pipes, water pipes, hookahs, and e-cigarettes among youths, these findings highlight the importance of also continuing to use targeted evidence-based, population-level strategies to combat combustible product use. These strategies include tobacco price increases, high-impact antitobacco mass media campaigns, comprehensive smoke-free laws, and enhanced access to help quitting tobacco to reduce smoking-related death and disease in the United States (*1*).

Observed disparities in tobacco product use across population groups likely have multiple contributing factors. For example, disparities in tobacco use by race/ethnicity might be partly explained by socio-cultural influences, norms surrounding the acceptability of tobacco use, and targeted marketing (*1*,*4*). Differences by education might be partly attributable to variations in understanding of the range of health hazards caused by tobacco product use (*1*,*4*). Differences by health insurance coverage and income might be attributable in part to variations in tobacco cessation coverage across insurance types and access to evidence-based cessation treatments, respectively (*1*,*5*). Furthermore, the higher prevalence of current tobacco product use among persons who identified as LGB might be due, in part, to social stressors including stigma and discrimination, in addition to targeted marketing efforts by the tobacco industry (*1*,*6*). Similarly, the higher rates of pipe, water pipe, hookah, and e-cigarette use among younger adults could be due to the manner in which these products are marketed and used socially (*1*,*7*). The tobacco industry has targeted marketing toward minority communities, persons of lower socioeconomic status, and younger persons (*4*,*6*). Lastly, the high prevalence of tobacco use among persons with serious psychological distress possibly reflects nicotine’s stimulant or relaxation effects, nicotine’s effects on drug metabolism, misperceptions about quitting smoking and abstinence success, and allowing smoking in mental health facilities (*4*,*8*).

The findings in this report are subject to at least three limitations. First, tobacco use estimates were self-reported and not validated by biochemical tests. However, previous studies have shown that self-reported tobacco product use is highly correlated with serum cotinine levels (*9*,*10*). Second, the NHIS response rate (55.2%) could introduce nonresponse bias if respondents and nonrespondents systematically differ in ways not accounted for in the development of the weights. Finally, NHIS does not include institutionalized populations and persons in the military, so the results are not generalizable to those groups.

Sustained, comprehensive state tobacco control programs can accelerate progress toward reducing tobacco-related diseases and deaths.^§§^ Full implementation of comprehensive tobacco control programs, in conjunction with FDA regulation of tobacco products, across the spectrum of tobacco products, are vital (*1*). Targeted interventions are also warranted to reach subpopulations with the greatest burden of use, which might vary by tobacco product type.

**Summary**

**What is already known about this topic?**

Tobacco use continues to be the leading cause of preventable disease and death in the United States. Despite declining cigarette smoking prevalence among U.S. adults, notable shifts in the tobacco product landscape have occurred in recent years.

**What is added by this report?**

In 2015, 20.1% of U.S. adults currently (every day or some days) used any tobacco product, 17.6% used any combustible tobacco product, and 3.9% used ≥2 tobacco products. Current use of any tobacco product was higher among males; persons aged <65 years; non-Hispanic American Indian/Alaska natives, whites, blacks, and persons of multiple races; persons living in the Midwest; persons with a General Educational Development certificate; persons with annual household income <$35,000; persons who were single/never married/not living with a partner or divorced/separated/widowed; persons who were insured through Medicaid or uninsured; persons with a disability; and persons who identified as lesbian, gay, or bisexual. Current use of any tobacco product was 47.2% among adults with serious psychological distress compared with 19.2% among those without serious psychological distress.

**What are the implications for public health practice?**

Full implementation of comprehensive tobacco control programs, in conjunction with FDA regulation of tobacco products, are vital across the spectrum of tobacco products. Targeted interventions are also warranted to reach subpopulations with the greatest burden of use, which might vary by tobacco product type.

## Footnotes

* Because of phrasing of the question in the 2015 NHIS, it was not possible to distinguish between regular pipe use and water pipe or hookah use in this analysis.

† *Northeast*: Connecticut, Maine, Massachusetts, New Hampshire, New Jersey, New York, Pennsylvania, Rhode Island, and Vermont. *Midwest:* Illinois, Indiana, Iowa, Kansas, Michigan, Minnesota, Missouri, Nebraska, North Dakota, Ohio, South Dakota, and Wisconsin. *South:* Alabama, Arkansas, Delaware, District of Columbia, Florida, Georgia, Kentucky, Louisiana, Maryland, Mississippi, North Carolina, Oklahoma, South Carolina, Tennessee, Texas, Virginia, and West Virginia. *West*: Alaska, Arizona, California, Colorado, Hawaii, Idaho, Montana, Nevada, New Mexico, Oregon, Utah, Washington, and Wyoming.

§ Sexual orientation was determined based on the question, “Which of the following best represents how you think of yourself?” with response options of “gay” (“lesbian or gay” for female respondents), “heterosexual,” that is, “not gay” (“not lesbian or gay” for female respondents), “bisexual,” “something else,” and “I don’t know the answer.” Responses were considered to be “LGB” if persons responded “gay,” “lesbian or gay,” or “bisexual.”

¶ Private coverage: includes adults who had any comprehensive private insurance plan (including health maintenance organizations and preferred provider organizations). Medicaid: for adults aged <65 years, includes adults who do not have private coverage, but who have Medicaid or other state-sponsored health plans including Children’s Health Insurance Program (CHIP); for adults aged ≥65 years, includes adults aged ≥65 years who do not have any private coverage but have Medicare and Medicaid or other state-sponsored health plans including CHIP; Medicare only: includes adults aged ≥65 years who only have Medicare coverage; Other coverage: includes adults who do not have private insurance, Medicaid, or other public coverage, but who have any type of military coverage, coverage from other government programs, or Medicare. Uninsured: includes adults who have not indicated that they are covered at the time of the interview under private health insurance, Medicare, Medicaid, CHIP, a state-sponsored health plan, other government programs, or military coverage.

** Disability was defined based on self-reported presence of selected limitations including vision, hearing, cognition, and movement. Limitations in performing activities of daily living were defined based on response to the question, “Does [person] have difficulty dressing or bathing?” Limitations in performing instrumental activities of daily living were defined based on response to the question, “Because of a physical, mental, or emotional condition, does [person] have difficulty doing errands alone such as visiting a doctor’s office or shopping?” Any disability was defined as a “yes” response pertaining to at least one of the limitations listed (i.e., vision, hearing, cognition, movement, activities of daily living, or instrumental activities of daily living). A random sample of half of the respondents from the 2015 Person File were asked about limitations.

†† The Kessler psychological distress scale is a series of six questions that ask about feelings of sadness, nervousness, restlessness, worthlessness, and feeling like everything is an effort in the past 30 days. Participants were asked to respond on a Likert Scale ranging from “None of the time” (score = 0) to “All of the time” (score = 4). Responses were summed over the six questions; persons with a score of ≥13 were coded as having serious psychological distress, and respondents with a score <13 were coded as not having serious psychological distress.

§§
https://www.cdc.gov/tobacco/stateandcommunity/best_practices/pdfs/2014/comprehensive.pdf .
